# PROTOCOL: Criminal justice interventions for preventing terrorism and radicalisation: An evidence and gap map

**DOI:** 10.1002/cl2.1273

**Published:** 2022-09-01

**Authors:** Michelle Sydes, Lorelei Hine, Angela Higginson, Laura Dugan, Lorraine Mazerolle

**Affiliations:** ^1^ School of Criminology and Criminal Justice Griffith University Brisbane Australia; ^2^ School of Social Science University of Queensland Brisbane Australia; ^3^ School of Justice, Faculty of Law Queensland University of Technology Brisbane Australia; ^4^ Department of Sociology The Ohio State University Columbus Ohio USA; ^5^ School of Social Science Univeristy of Queensland Brisbane Australia

## Abstract

This is the protocol for a Campbell systematic review. The objectives are as follows: to identify the existing evidence that considers the effectiveness of criminal justice interventions in preventing terrorism and radicalisation and to identify existing gaps in the evidence where new primary research could be undertaken and where future synthesis could be conducted.

## 1 BACKGROUND

### 1.1 The problem, condition or issue

Violent radicalisation and terrorism continue to represent an ongoing threat to global security, with 44 countries experiencing at least one death from terrorism in the past year (Institute for Economics and Peace IEP, 2022). While definitions vary (Borum, 2015; Desmarais et al., 2017; Horgan, 2008; Kruglanski et al., 2019; Sarma, 2017) violent radicalisation is broadly defined as the process by ‘which a person adopts extremist views and moves towards committing a violent act’ (Hardy, 2018; p. 76; see also Irwin, 2015; Jensen et al., 2018). Once radicalised, individuals and groups *may* go on to carry out terrorist attacks against innocent civilians, key infrastructure, symbolic targets or specific groups (Wilner & Dubouloz, 2010) or travel to conflict zones to engage in violence with extremist groups (Lindekilde et al., 2016). Preventing violent radicalisation has thus become a key focus of counterterrorism agendas globally (Dudenhoefer, 2018; Elshimi, 2017; Heath‐Kelly, 2013).

The harms associated with violent radicalisation and terrorism are far‐reaching. Between 2009 and 2019, violent radicalisation and terrorism were listed as the cause of 21,000 deaths on average per year, with the majority occurring in the Middle East, Africa or South Asia (Ritchie et al., 2019). The estimated economic impact of terrorism is similarly staggering. According to Bardwell and Iqbal (2020); the cost of terrorism to the world economy between 2000 and 2018 was 855 billion USD. Beyond the human casualties and economic consequences, violent radicalisation and terrorism are linked to a broad range of psychological, social and political impacts. Studies find experiences of terrorism can have negative mental health implications particularly among first responders and victims who face high levels of trauma and depression in the aftermath (Razik et al., 2013; Salguero et al., 2011). Violent radicalisation and terrorism can also antagonise public attitudes towards immigration and groups perceived as a terrorist threat (Williamson & Murphy, 2020). Right‐wing political parties frequently manipulate fear of terrorism and violent radicalisation to garner support for hard‐line and discriminatory counterterrorism policies (Haner et al., 2019, 2021; Williamson, 2019). Such policies often result in the over‐surveillance of ‘suspect communities’ (Breen‐Smyth, 2014; Cherney & Murphy, 2016), further isolating groups at risk of radicalisation and in turn, providing motivation for retaliatory attacks (Fisher et al., 2019; Jacobs & van Spanje, 2021).

Research suggests that individual pathways to radicalisation can be influenced by a broad range of factors including social networks, affiliation with certain political organisations, places of worship, familial relationships or exposure to radical individuals during incarceration. These predictors can also interact with personal factors such as psychological disorders or traumatic experiences, further heightening the risk of radicalisation (Jensen et al., 2018; Kruglanski et al., 2019). Effectively intervening to prevent and reduce the likelihood of violent radicalisation and terrorism is therefore often difficult due to the significant variation in the predictors of violent radicalisation amongst individuals and extremist groups (Desmarais et al., 2017; Wolfowicz et al., 2019).

### 1.2 The intervention

Criminal justice agencies (i.e., police, courts and corrections) are well positioned to help prevent the radicalisation of individuals and groups, stop those radicalised from engaging in violence, and reduce the likelihood of terrorist attacks. Following September 11, police became more actively involved in countering terrorism ‐ disrupting terrorist activity through intelligence gathering, covert investigations, information sharing and enforcement activities (Ortiz et al., 2007). As frontline practitioners, police are also instrumental in identifying, reducing, and preventing both radicalisation and violent extremism (Mazerolle et al., 2021). Police approaches for preventing radicalisation and violent extremism involve a range of different interventions and strategies. These include police facilitating training programs to improve officer recognition and responses to radicalisation or police delivering awareness programs to educate others on how to identify and report radicalised individuals (Carter, 2014; Davis et al., 2016). Some police agencies also work with communities to build resilience to protect against the influence of extremist messages and recruiters (Cherney & Hartley, 2017; Mazerolle et al., 2020; Schanzer et al., 2016). Such initiatives focus on bolstering community engagement, trust and connectedness while reducing feelings of social isolation (Mazerolle et al., 2020).

The courts and correctional agencies also play a critical role in preventing terrorism and radicalisation. Court officials may direct radicalised individuals or individuals who have engaged in violent extremism and/or terrorist activity to various support services or mandate participation in rehabilitation programs designed to counter violent extremism (Cherney et al., 2021). Prisons are widely believed to provide a prime opportunity for radicalisation to flourish, threatening the safety and security of both inmates and the community more broadly (van der Heide & Kearney, 2020). Training initiatives and awareness programs with corrective services staff may assist in the detection and prevention of both radicalisation and violent extremism (Vejvodová & Kolář, 2020). In an attempt to prevent the spread of radicalised ideologies behind bars, corrective services agencies often segregate radicalised individuals from the general prison population (Jones & Narag, 2018). Indeed, some jurisdictions operate specialised terrorist units to house extremist offenders (van der Heide & Kearney, 2020), with the aim to prevent radicalised inmates from influencing other prisoners and allow staff interacting with radicalised inmates to be upskilled in best practice approaches. Corrective services agencies may also design and deliver deradicalisation programs to radicalised inmates or inmates convicted of terror‐related offences (Cherney & Belton, 2021). Upon release, community corrections staff may provide radicalised offenders with the support necessary for successful reintegration back into the community (Cherney, 2021).

Recognising the complexities surrounding radicalisation and terrorism, many criminal justice agencies are increasingly taking an intersectoral and multi‐agency approach. Such approaches acknowledge the difficulties a single agency or organisation may face in trying to combat the problem in isolation (Mazerolle et al., 2021). These initiatives include promoting information sharing between agencies (Knight, 2009), community outreach activities (Lamb, 2013; Schanzer et al., 2016), intelligence gathering (Lewandowski, 2012) and training programs (Davis et al., 2016; Kerry, 2007). For example, the World Organisation for Resource Development Education (WORDE) program which was led by a non‐profit group of Muslim scholars and community leaders, partnered with several agencies including law enforcement. The program comprised three key components: (1) community education via town hall meetings; (2) the development of a referral network for high‐risk individuals; and (3) participation in community activities (Williams et al., 2016). By partnering with other agencies and community groups, the intervention aimed to tackle the problem of radicalisation in a multi‐faceted manner (Beutel & Weinberger, 2016; Mazerolle et al., 2021).

Criminal justice interventions to prevent terrorism and violent radicalisation thus cover a broad range of policies and programs (Hardy, 2020). Prevention efforts can be divided into primary prevention (interventions targeted at a population), secondary prevention (interventions targeted at high‐risk individuals) and tertiary prevention (interventions targeted at already radicalised individuals) (Harris‐Hogan et al., 2015). Criminal justice interventions which respond to terrorism/radicalisation are thus delivered across a variety of settings with participants ranging from community members to convicted terrorists (Gielen, 2019). As such, there is no singular theoretical framework underpinning criminal justice interventions for preventing terrorism and radicalisation. Prevention efforts by criminal justice agencies vary considerably in their underlying mechanisms and intended outcomes (Hardy, 2020). For instance, primary intervention programs may focus on building community capacity/cohesion and feelings of inclusion (Mazerolle et al., 2020), secondary prevention programs may aim to reduce vulnerability or risk of radicalisation (Jones & Narag, 2018) and tertiary prevention programs may seek to deradicalise convicted extremists (Cherney & Belton, 2021).

### 1.3 Why it is important to develop the EGM

Built using data collected via systematic search methods, EGMs provide a visual display of the available research evidence related to a particular topic area (White et al., 2020). EGMs are typically presented as a matrix comprising intervention categories (rows) and outcomes (columns). Interactive EGMs may offer additional filters which allow users to view the information based on certain classifications (e.g., study location, document type, study quality) (White et al., 2020). By locating and visually presenting all available evidence, EGMs improve stakeholder access to impact evaluations. While EGMs are a relatively new method of evidence synthesis, they are increasingly used to map evidence related to crime and justice issues (see e.g., Eggins et al., 2021; Pundir et al., 2020).

There are currently no existing EGMs that focus on criminal justice responses to radicalisation, violent extremism, and/or terrorism. Several systematic reviews have synthesised the effectiveness literature. However, these reviews have either had a broad scope including criminal justice agencies amongst other agencies (Lum et al., 2006) or a more targeted scope focusing on just a particular branch of the criminal justice system (see e.g., Mazerolle et al., 2021 for multi‐agency responses to radicalisation with police as a partner). Other reviews have focused on a particular type of program. For example, Mazerolle et al. (2020) reviewed police programs that seek to increase community connectedness for reducing violent extremism behaviour, attitudes and beliefs. Our systematic search will include all types of criminal justice interventions that prevent or respond to radicalisation, violent extremism and/or terrorism. It is expected that this EGM will help promote the translation of evidence into practice by providing practitioners and policymakers with easy access to evaluation evidence.

This EGM will be used to demonstrate gaps in the evaluation research, allowing key stakeholders to identify future funding priorities for research and development. Specifically, if the EGM indicates a lack of research related to a particular intervention/outcome, this may provide grounds to justify future primary studies. Additionally, if the EGM illustrates several studies in one domain, this may lead to further syntheses such as meta‐analysis.

## 2 OBJECTIVES

This EGM aims to provide a comprehensive and systematic display of the existing evidence on criminal justice interventions which aim to prevent and reduce terrorism and radicalisation. It will map the extant evaluation evidence across the policing, courts, and corrections arms of the criminal justice system including multi‐agency responses. The key objectives of this EGM are as follows:

1. To identify the existing evidence that considers the effectiveness of criminal justice interventions in preventing terrorism and radicalisation.
2. To identify existing gaps in the evidence where new primary research could be undertaken and where future synthesis could be conducted.

## 3 METHODS

### 3.1 Evidence and gap map: Definition and purpose

EGMs provide an interactive and graphical representation of the available evidence concerning a particular topic area (Snilstveit et al., 2016). EGMs are usually presented as a matrix with interventions displayed in rows and outcomes presented in columns (White et al., 2020). Like systematic reviews, EGMs utilise rigorous systematic search methods (White et al., 2020). However, EGMs are usually broader in scope than systematic reviews. EGMs are often viewed as ‘public goods’ as they improve acess to high‐quality evidence for policymakers, practitioners and the wider public (Snilstveit et al., 2016). Our EGM will help to highlight both the density and paucity of existing research by including both primary studies and systematic reviews related to criminal justice interventions for preventing and responding to terrorism and radicalisation.

### 3.2 Framework development and scope

The framework used in the EGM will be both theoretically and empirically informed. The initial framework to design inclusion and exclusion criteria and search strategies is based on an a priori schema, described below, and will be informed by the practical experiences and academic knowledge of the Advisory Group. Building on this framework, we will conduct a thematic cluster analysis of the included studies to develop an empirical classification schema for the final EGM categories and domains.

### 3.3 Stakeholder engagement

Initial stakeholder consultation took place with the Department of Homeland Security in July 2021 to determine the outcome categories of interest and EGM scope. We will further consult a Virtual Advisory Group comprised of key experts in the field and government stakeholders. We have invited stakeholders from various government agencies across the United States, United Kingdom, Australia, New Zealand, Canada and Sweden to join our Advisory Group. We have additionally invited researchers from Spain, Israel, the United States and Australia. When finalised, our Advisory Group will provide a diverse range of perspectives regarding criminal justice responses to terrorism/radicalisation.

Due to varied time zones, we will continue to contact Advisory Group members via email. As part of their role, members of the Advisory Group have been asked to provide feedback on search locations, recommend potentially eligible studies and help to locate hard to reach documents. Advisory Group members will additionally be offered the opportunity to review and provide feedback on the final draft report. A list of all represented agencies on the Advisory Group will be provided in the final report.

### 3.4 Dimensions

Our EGM will be presented as a matrix of interventions (rows) and outcomes (columns). The number of primary studies will be shown by the size of the bubble. The proposed dimensions of the map are as follows:

- Row headings: Intervention—two layers for intervention agency and intervention category
  o Intervention agency (police, courts, custodial corrections, community‐based corrections, multi‐agency with one or more criminal justice partner) and
  o Intervention category (e.g., training, community awareness, cognitive behavioural therapy, prisoner isolation, etc.)
- Column headings: Outcome category
  o Extremism, radicalisation indicators (e.g., Violent Extremist Risk Assessment‐2, Extremist Risk Guidance Factors, IAT‐8, RADAR assessments, Terrorist Radicalisation Assessment Protocol)
  o Crime or recidivism (e.g., arrests, sentences, calls‐for‐service, re‐offending)
  o Other (e.g., psychosocial, perceptions of trust, perceptions of legitimacy)

#### 3.4.1 Types of study design

Systematic reviews and randomised controlled trials (RCTs) are considered the gold standard for ascertaining intervention effectiveness. Eligible comparison conditions/groups for inclusion in the EGM will include no treatment, placebo, ‘business as usual,’ waitlist control, or an alternative treatment. While other designs are less causally robust, they may be appropriate to include due to the difficulties associated with conducting RCTs in criminal justice settings, particularly when targeting radicalisation, violent extremism, and/or terrorism. The strong quasi‐experimental designs eligible for this EGM include:

- Matched control group designs with or without preintervention baseline measures (propensity or statistically matched)
- Unmatched control group designs with pre–post intervention measures that allow for difference‐in‐difference analysis
- Unmatched control group designs without preintervention baseline measures where the control group has face validity
- Regression discontinuity designs
- Cross‐over designs
- Designs using multivariate controls (e.g., multiple regression)
- Short interrupted time series designs with control group (<25 preintervention and 25 postintervention observations (Glass, 1997)
- Long interrupted time series designs with or without a control (≥25 preintervention and postintervention observations (Glass, 1997).

In line with previous reviews (Mazerolle et al., 2020; Mazerolle et al., 2021) which recognise the significant shift in counterterrorism policies post 9/11 (Gaibulloev & Sandler, 2019), only studies that are published from or report on impact evaluations conducted between January 2002 and December 2021 will be included in the EGM. We will not include qualitative research as we are exclusively interested in mapping high quality impact evaluations. Should our searches identify any ongoing studies that meet our selection criteria but for which findings have not yet been published, we will include these as ongoing studies. These will be assessed for possible inclusion when the review is updated by the authors.

#### 3.4.2 Types of intervention/problem

Our EGM will capture criminal justice interventions that aim to prevent or respond to radicalisation, violent extremism, and/or terrorism.

A *criminal justice intervention* is defined as a strategy, technique, approach, activity, campaign, training, directive, funding, or organisational change that involves at least one criminal justice agency. Criminal justice agencies include police, courts and corrections (both custodial and community based). Interventions that involve partnerships between at least one criminal justice agency and at least one external agency/organisation will also be included. (Eggins et al., 2021; Mazerolle et al., 2018). Examples of criminal justice interventions may include:

- Criminal justice system initiation, development, or leadership of the intervention.
- Criminal justice system staff or populations as recipients of the intervention.
- Criminal justice system practices as the focus or target of the intervention.
- The criminal justice system delivers or implements the intervention.

*Radicalisation* is defined as the process of an individual or group adopting extreme political, social, or religious ideals (Hardy, 2018; Jensen et al., 2018). Analogous concepts are *disengagement* and/or *deradicalisation*, which are often encompassed within conceptualisations of violent extremism. *Disengagement* refers to reducing or ceasing physical involvement in violent or radical activities, while *deradicalisation* is defined as the psychological shift in attitudes or beliefs (Windisch et al., 2016).

*Violent extremism* is defined as ‘advocating, engaging in, preparing, or otherwise supporting ideologically motivated or justified violence to further social, economic, and political objectives’ (US Agency for International Development, 2016).

*Terrorism* is defined as ‘the unlawful use of force or violence against persons or property to intimidate or coerce a government, the civilian population or any segment thereof, in furtherance of political or social objectives’ (Shanahan, 2016, p. 108).

Eligible interventions may include but are not limited to:

- Police training programs to improve officer recognition and responses to radicalisation and terrorism (Davis et al., 2021).
- Police working with communities to build resilience to protect against the influence of extremist messages and recruiters (Cherney & Hartley, 2017; Mazerolle et al., 2020; Schanzer et al., 2016).
- Court officials mandating participation in rehabilitation programs designed to counter violent extremism (Cherney et al., 2021).
- Corrections agencies installing terrorist wings within prisons to separate radicalised prisoners or prisoners convicted of terrorist acts from the general prison population (van der Heide & Kearney, 2020).
- Corrections agencies delivering specialised and individualised rehabilitation programs designed to challenge radicalised views (Cherney & Belton, 2021).
- Training initiatives and awareness programs with corrective services staff focused on the detection and prevention of both radicalisation and violent extremism (Vejvodová & Kolář, 2020).
- Re‐entry programs focused on building pro‐social networks and access to support services to assist with prisoner release (Cherney, 2021).
- Multi‐agency initiatives that promote information sharing and intelligence gathering between agencies (Knight, 2009; Lewandowski, 2012).
- Multi‐agency initiatives such as community outreach activities (Lamb, 2013; Schanzer et al., 2016).
- Multi‐agency initiatives focused on delivering training to various stakeholders on how to identify and report radicalised individuals (Carter, 2014; Davis et al., 2016; Kerry, 2007).

Eligible comparison conditions/groups for inclusion in the EGM will include no treatment, placebo, ‘business as usual,’ waitlist control, or an alternative treatment. If a study reports on multiple interventions whereby only a subset meets the eligibility criteria, only the eligible interventions will be included in the map.

#### 3.4.3 Types of population

The following populations will be included in the EGM:

- Criminal justice practitioners
- Victims
- Communities
- Individuals or groups who have been identified as at risk of becoming radicalised or engaging in violent extremism and/or terrorist activity
- Radicalised individuals or groups (including pre‐criminal justice involved radicalised individuals)
- Individuals or groups who have engaged in violent extremism and/or terrorist activity
- Family members of radicalised individuals or individuals who have engaged in violent extremism and/or terrorist activity
- Micro places (e.g., street corners, buildings, police beats, street segments)
- Macro places (neighbourhoods or larger geographies)

#### 3.4.4 Types of outcome measures

No restrictions will be placed on the types of outcomes used to evaluate criminal justice responses for preventing radicalisation, violent extremism and/or terrorism. Depending on the studies identified, we tentatively plan to group outcomes under three domains:

1. Extremism/radicalisation indicators such as
  a. Violent Extremist Risk Assessment‐2 (VERA‐2): a risk assessment tool used to assess an individual's risk of violent extremism (Pressman & Flockton, 2013)
  b. Extremist Risk Guidance Factors (ERG 22+): a structured professional judgement (SPJ) which assesses ‘the risks, needs, and vulnerabilities to be managed to prevent offending’ (Her Majesty's Inspectorate of Probation, 2021).
  c. RADAR assessments: a risk assessment tool that identifies ‘high‐risk individuals who would benefit from programs designed to prevent radicalisation’ (RTI International, 2018 p. 19).
  d. IAT‐8: a risk assessment tool that considers whether an intervention is influencing ‘'the level of vulnerability to radicalisation’ (RTI International, 2018 p. 10).
  e. Terrorist Radicalisation Assessment Protocol: a SJP instrument used to identify individuals at risk of lone‐actor terrorism. The tool is intended to be used by ‘mental health, intelligence, law enforcement and security professionals to manage operational data on a person of concern and prioritise cases based upon the presence or absence of warning behaviours and characteristics’ (Meloy, 2016)
2. Crime and recidivism measures. These include:
  a. Crime
    i. Official records (e.g., arrests, charges and convictions for both terror and non‐terror related offences)
    ii. Self‐reported offending behaviour
    iii. Perceptions of crime/disorder
  b. Recidivism
    i. Technical violations of parole conditions
    ii. Any contact with the criminal justice system for a new offence.
3. Other:
  a. Should a pattern emerge in the outcomes initially categorised as ‘other', we will group these together under an appropriate label (e.g., psychosocial, perceptions of police).

Unintended or adverse outcomes will be included.

##### Types of location/situation

We will place no limits on the geographical region reported in the study. No restrictions will be placed on the language a document is written in. Similar to other reviews (e.g., Mazerolle et al., 2020; Davis et al., 2021), titles/abstracts written in a language other than English will be translated using Google Translate to determine if they are potentially eligible for the EGM. We will likewise translate portions of full‐text documents in a language other than English using Google Translate to ascertain eligibility. If we cannot determine eligibility through this approach, we will contact study authors to determine eligibility.

##### Types of settings

No restrictions will be placed on the settings used in eligible studies.

### 3.5 Search methods and sources

#### 3.5.1 Global policing database

The search for this review will be led by the Global Policing Database (GPD) research team at the University of Queensland (Lorraine Mazerolle and Lorelei Hine), Queensland University of Technology (Angela Higginson) and Griffith University (Elizabeth Eggins). The University of Queensland is home to the GPD (see http://www.gpd.uq.edu.au), which will serve as a key search location for this review. The GPD is a web‐based and searchable database designed to capture all published and unpublished experimental and quasi‐experimental evaluations of policing interventions conducted since 1950. There are no restrictions on the type of policing technique, type of outcome measure or language of the research. The GPD is compiled using systematic search and screening techniques, which are reported in Higginson et al., (2015) and summarised in Supporting Information: Appendices 1 and 2. Broadly, the GPD search protocol includes an extensive range of search locations to ensure that both published and unpublished research is captured across criminology and allied disciplines.

To capture studies for this review, we will use terms related to terrorism and radicalisation to search the GPD corpus of full‐text documents that have been screened as reporting on a quantitative impact evaluation of a policing intervention. Specifically, we will use the following terms to search the title and abstract fields of the corpus of documents published between 1 January 2002 and 31 December 2021:

extremis* OR ‘far#left*’ OR ‘far#right*’ OR ‘foreign#fight*’ OR ‘freedom#fight*’ OR guerrilla OR ‘homeland#security’ OR ‘ideological violence*’ OR ‘ideologically#motivat*’ OR indoctrinat* OR ‘left#wing*’ OR ‘lone#wol*’ OR militant* OR ‘national#security’ OR ‘political violence*’ OR ‘politically#motivat*’ OR radicali* OR rebel* OR ‘religious violence*’ OR ‘religiously#motivat*’ OR ‘right#wing*’ OR ‘single#issue*’ OR supremacis* OR terror* OR vigilante* OR vigilantism OR deradicali* OR ‘de‐radicali*’ OR ‘counter‐terror*’ OR counterterror* OR ‘counter‐extremis*’ OR counterextremis* OR separatis* OR militia* OR jihad*

#### 3.5.2 Other database searches

To capture interventions in the courts, corrections and multi‐agency spheres not encapsulated within the GPD, we will conduct searches of key academic sources in criminology and criminal justice (see Table 1). We will search these databases on three sets of terms across title, abstract, keyword, and/or subject/indexing fields. These aim to capture literature on (1) terrorism within the (2) criminal justice system that uses a term indicative of a (3) evaluation of an intervention. Terms within each set will be combined with Boolean OR operators, and the three sets will then be combined with Boolean AND operators (see Supporting Information: Appendix 3 for an example search syntax). This search approach has been informed by Eggins et al. (2021) systematic review of criminal justice system responses to child sexual abuse material offending. We will search the following across the title, abstract, keyword, and/or subject/indexing fields (adapting the search to the database if fields are unavailable):

(1) extremis* OR ‘far#left*’ OR ‘far#right*’ OR ‘foreign#fight*’ OR ‘freedom#fight*’ OR guerrilla OR ‘homeland#security’ OR ‘ideological violence*’ OR ‘ideologically#motivat*’ OR indoctrinat* OR ‘left#wing*’ OR ‘lone#wol*’ OR militant* OR ‘national#security’ OR ‘political violence*’ OR ‘politically#motivat*’ OR radicali* OR rebel* OR ‘religious violence*’ OR ‘religiously#motivat*’ OR ‘right#wing*’ OR ‘single#issue*’ OR supremacis* OR terror* OR vigilante* OR vigilantism OR deradicali* OR ‘de‐radicali*’ OR ‘counter‐terror*’ OR counterterror* OR ‘counter‐extremis*’ OR counterextremis* OR separatis* OR militia* OR jihad*
  AND
(2) accused OR acquit* OR adjourn* OR adjudicat* OR *admiss* OR affida* OR appeal* OR appellate OR apprehend* OR arbitrat* OR arraign* OR *arrest* OR attorney* OR authorit* OR bail* OR barrister* OR breach* OR ‘case#manage*’ OR caution* OR charge* OR clerk* OR confinement* OR convict* OR coroner* OR correction* OR court* OR crime* OR criminal* OR ‘cross#examin*’ OR custod* OR defendant* OR defense OR defence OR detain* OR detention* OR deter* OR divert* OR diversion* OR enforc* OR execut* OR felon* OR forensic* OR gaol* OR guilt* OR ‘high#security’ OR ‘halfway#house’ OR *imprison* OR incarcerat* OR indict* OR infract* OR infring* OR injunct* OR inquest* OR innocen* OR inmate* OR juris* OR jail* OR judge* OR judic* OR juror* OR juries OR jury OR justice OR law* OR legal* OR legislat* OR litigat* OR ‘low#security’ OR magistrate* OR mandat* OR mitigat* OR marshal* OR misdem* OR ‘medium#security*’ OR offend* OR offence* OR officer* OR official* OR ordinance OR parole* OR pardon* OR penal* OR plea* OR precedent* OR prevent* OR prison* OR probat* OR prohibit* OR prosecut* OR punish*OR recividis* OR rehab* OR reintegrat* OR remand* OR reoffend* OR ‘re‐offend*’ OR ruling* OR sanction* OR sentenc* OR solicitor* OR statut* OR subpoena* OR supervis* OR surveil* OR suspect* OR testif* OR testimon* OR *trial* OR tribunal* OR verdict* OR victim*OR witness*
  AND
(3) ‘comparison#condition*’ OR ‘comparison#group*’ OR ‘control#condition*’ OR ‘control#group*’ OR effective OR efficac* OR evaluat* OR experiment* OR intervent* OR ‘matched#group*’ OR program* OR ‘quasi#experiment*’ OR random* OR RCT OR treatment* OR trial*

**Table 1:** Search plan

Source	Type	Approach	Website (if applicable)
Global Policing Database	Database	Terms: Terrorism terms only	
		Fields: Title, abstract	
EBSCO	Database	Terms: Full search string	
Criminal Justice Abstracts		Fields: Title, abstract, keywords	
HeinOnline	Database	Terms: Full search string	
Law Journal library		Fields: Text, title, subjects	
Criminal Justice & Criminology			
Foreign and International Law Resources Database			
United Nations Law Collection			
Informit	Database	Terms: Full search string with databases searched simultaneously	
Australian Criminology DB (CINCH)		Fields: Title, abstract, subjects	
AGIS Plus Text			
National Criminal Justice Reference Service	Database	Terms: We have access to the free website where we will conduct basic keyword searches for terrorism terms. Will be able to web‐scrape results.	
		Fields: General search, title	
ProQuest	Database	Terms: Full search string with databases searched simultaneously	
Criminal Justice		Fields: Title, abstract, keywords, subject	
Dissertations & These Global			
Digital National Security Archive			
PTSDPubs			
Social Science Database			
Sociological Abstracts (incl. Social Services Abstracts)			
FORENSICnetBASE	Database	Terms: Modified search due to database functionality, focus on terrorism search terms	
		Fields: Title, keyword	
OVID	Database	Terms: Full search string	
PsycINFO		Fields: Title, abstract, keywords, subject	
PsycEXTRA			
Web of Science	Database	Terms: Full search string	
Social Science Citation Index		Fields: Title, abstract, keywords, subject	
Conference Proceedings Index (Social Sciences & Humanities)			
Emerging Sources Index			
SciELO Citation Index			
MEDLINE			
Cochrane Library (including Cochrane Central Register of Controlled Trials (CENTRAL))	Database	Terms: Full search string	https://www.cochranelibrary.com/search
		Fields: Title, abstract, keywords	
World Health Organization International Trial Registry	Database	Terms: Full search string	https://trialsearch.who.int/
		Fields: Title, abstract, keywords	
NIH RePORTER (clinical studies)	Database	Terms: Modified search due to database functionality, focus on terrorism search terms	https://reporter.nih.gov/
		Fields: Title, abstract, keywords	
Trials Register of Promoting Health Interventions (TRoPHI)	Database	Terms: Modified search due to database functionality, focus on terrorism search terms	https://eppi.ioe.ac.uk/webdatabases4/SearchIntro.aspx
		Fields: Title, abstract, keywords	
AEA RCT Registry	Database	Terms: Modified search due to database functionality, focus on terrorism search terms	https://www.socialscienceregistry.org/
		Fields: Title, abstract, keywords	
CrimeSolutions.Gov	Grey	Hand search for terrorism intervention literature in the corrections subsets	https://crimesolutions.ojp.gov/
Urban Institute	Grey	Hand search for terrorism intervention literature	https://www.urban.org/policy-centers/justice-policy-center/publications
What Works Toolkit	Grey	Hand search for terrorism intervention literature	http://whatworks.college.police.uk/toolkit/Pages/Toolkit.aspx
National Institute of Corrections	Grey	Hand search for terrorism intervention literature	https://nicic.gov/
RAND	Grey	Hand search for terrorism intervention literature in the Better Policing Toolkit, the Courts and Corrections sections	https://www.rand.org/pubs/tools/TL261/better-policing-toolkit.html
			https://www.rand.org/jie/justice-policy/pubs/courts.html
			https://www.rand.org/jie/justice-policy/correctional-education.html
			https://www.rand.org/jie/justice-policy/projects/priority-criminal-justice-needs.html
UK Government research	Grey	Hand search for terrorism intervention literature. Policing literature is already captured in GPD for this site, so the search will target the courts/corrections agencies	https://www.gov.uk/search/research-and-statistics
Prison Research Centre	Grey	Hand search for terrorism intervention literature	https://www.prc.crim.cam.ac.uk/publications/articles
Correctional Service Canada	Grey	Hand search for terrorism intervention literature	http://www.csc-scc.gc.ca/research/005008-2006-eng.shtml
NZ Corrective Services	Grey	Hand search for terrorism intervention literature	http://www.corrections.govt.nz/resources/research_and_statistics.html
Centre for Advancing Correctional Excellence	Grey	Hand search for terrorism intervention literature	https://www.gmuace.org/
Victorian Corrections, Prisons and Parole	Grey	Hand search for terrorism intervention literature	https://www.corrections.vic.gov.au/publications-manuals-and-statistics
New South Wales Corrective Services	Grey	Hand search for terrorism intervention literature	http://www.correctiveservices.justice.nsw.gov.au/Pages/CorrectiveServices/related-links/publications-and-policies/corrections-research-evaluation-and-statistics/Research_Publication.aspx
			http://www.correctiveservices.justice.nsw.gov.au/Pages/CorrectiveServices/related-links/publications-and-policies/corrections-research-evaluation-and-statistics/collaborative-reports.aspx
South Australia Corrective Services	Grey	Hand search for terrorism intervention literature	https://www.corrections.sa.gov.au/about/our-research
Western Australia Corrective Services	Grey	Hand search for terrorism intervention literature	https://www.correctiveservices.wa.gov.au/about-us/statistics-publications/default.aspx
Northern Territory Corrective Services	Grey	Hand search for terrorism intervention literature	https://justice.nt.gov.au/attorney-general-and-justice/justice-publications
Tasmanian Corrective Services	Grey	Hand search for terrorism intervention literature	https://www.justice.tas.gov.au/
Australian Capital Territory Corrective Services	Grey	Hand search for terrorism intervention literature	http://www.cs.act.gov.au/act_corrective_services/stats_and_publications
Queensland Corrective Services	Grey	Hand search for terrorism intervention literature	https://corrections.qld.gov.au/documents/reviews-and-reports/
Global Terrorism Research Centre (Monash University)	Grey	Hand search for criminal justice intervention literature	https://www.monash.edu/arts/social-sciences/gtrec/publications
Triangle Centre on Terrorism and Homeland Security	Grey	Hand search for criminal justice intervention literature	https://sites.duke.edu/tcths/#
Department of Homeland Security	Grey	Hand search for criminal justice intervention literature	https://www.dhs.gov/topics
Public Safety Canada	Grey	Hand search for criminal justice intervention literature	https://www.publicsafety.gc.ca/index-en.aspx
National Consortium for the Study of Terrorism and Responses to Terrorism (START)	Grey	Hand search for criminal justice intervention literature	https://www.start.umd.edu/
Terrorism Research Centre	Grey	Hand search for criminal justice intervention literature	http://www.terrorism.org/
Global Centre on Cooperative Security	Grey	Hand search for criminal justice intervention literature	https://www.globalcenter.org/publications/
Hedayah	Grey	Hand search for criminal justice intervention literature	http://www.hedayahcenter.org/publications
Radicalisation Awareness Network (RAN)	Grey	Hand search for criminal justice intervention literature	https://ec.europa.eu/home-affairs/what-we-do/networks/radicalisation_awareness_network_en
RadicalisationResearch	Grey	Hand search for criminal justice intervention literature	https://www.radicalisationresearch.org/
Royal United Services Institute (RUSI)	Grey	Hand search for criminal justice intervention literature	https://rusi.org/
Impact Europe	Grey	Hand search for criminal justice intervention literature	http://impacteurope.eu/
International Association of Law Enforcement Intelligence Analysts	Grey	Hand search for terrorism intervention literature	https://www.ialeia.org/resources_publications.php
Naval Post‐Graduate School	Grey	Hand search for terrorism intervention literature	https://nps.edu/web/research/home
International Centre for Counter‐Terrorism	Grey	Hand search for criminal justice intervention literature	https://icct.nl/topic/criminal-justice-response/
International Centre for the Study of Radicalisation	Grey	Hand search for criminal justice intervention literature	https://icsr.info/publications/
Combating Terrorism Center	Grey	Hand search for criminal justice intervention literature	https://ctc.usma.edu/ctc-sentinel/
Centre of Excellence Defence Against Terrorism	Grey	Hand search for criminal justice intervention literature	https://www.tmmm.tsk.tr/research.html
Campbell Collaboration – terrorism reviews	Journal	Hand search for terrorism intervention reviews	https://onlinelibrary.wiley.com/journal/18911803
Targeted journal search within Jstor for terrorism journals:	Journal	Terms: Targeted search within journal in Jstor. Search on courts/corrections and evaluation terms	
Counter Terrorist Trends & Analysis (CTTA)		Fields: Title, abstract, keywords	
Prism			
International Journal of Peace Studies			
Journal of Security, Intelligence and Resilience Education	Journal	Hand search for criminal justice intervention literature	https://jsire.org/about/
Journal of Terrorism Research	Journal	Hand search for criminal justice intervention literature	https://cvir.st-andrews.ac.uk/articles/search/
The Journal of International Security Affairs	Journal	Hand search for criminal justice intervention literature	https://security-affairs.com/about/
Journal of 9/11 Studies	Journal	Hand search for criminal justice intervention literature	http://www.journalof911studies.com/J911S/articles/

#### 3.5.3 Searching other resources

In addition to the database searches, we will use the following search strategies:

- Searching government and non‐government websites related to terrorism and radicalisation research (see Table 1)
- Searching government and non‐government websites related to courts and corrections for research related to terrorism and radicalisation interventions (see Table 1)
- Searching journals related to terrorism and radicalisation (see Table 1)
- Reference harvesting eligible documents
- Forward citation searching eligible documents using Google Scholar
- Contacting our advisory group and identified experts in the field (see Stakeholder Engagement) to identify any potentially eligible studies that are not yet published

### 3.6 Analysis and presentation

#### 3.6.1 Report structure

The EGM will comprise the following sections: (1) executive summary; (2) background; (3) objectives (4) methods (5) results and (6) discussion. The executive summary will provide an overview of the EGM findings, detailing the number of studies captured in the review across the criminal justice system (police, courts, corrections and multi‐agency). The background section will detail the problem of radicalisation, violent extremism and terrorism, outlining why and how the criminal justice system provides a prime opportunity for intervention to reduce and prevent its occurrence.

The methods will provide information on the systematic search, including key search terms and search locations. The methods will also describe the screening and data extraction processes and software programs utilised. The results will provide an overview of the number of eligible studies captured by the systematic search, detailed in a PRISMA figure. Our results will also include our interactive EGM, providing a graphical representation of the available evidence. The discussion will reflect on both the paucity and density of research across different intervention and outcome domains. We will recommend areas for future research and synthesis, and implications for policy and practice.

Planned Tables and Figures:

- Figure: PRISMA
- Table: Search locations
- Table: EGM—primary studies (non‐interactive)
- Table: EGM—systematic reviews (non‐interactive)
- Table: online interactive EGM—with filters

#### 3.6.2 Filters for presentation

The results of our online interactive EGM will be presented as a matrix of rows (comprising two layers: (1) intervention agency; (2) intervention type) and columns (outcomes). We will additionally employ the following filters:

- Study type (toggle filter): research synthesis, research study
- Research design: RCT, strong quasi‐experimental
- Document type: published, unpublished
- Study location: country, region
- Country income group: low income, middle income, high income
- Implementation setting: prisons/correctional facilities, courts, community, school, workplace, places of worship, home, other
- The focus of intervention: preventing radicalisation, preventing terrorism, preventing both
- Prevention category: primary, secondary, tertiary
- Age of targets: youth, adults, both
- Target population:
  o Criminal justice practitioners
  o Victims
  o Communities
  o Individuals or groups who have been identified as at risk of becoming radicalised or engaging in violent extremism and/or terrorist activity
  o Radicalised individuals or groups (including pre‐criminal justice involved radicalised individuals)
  o Individuals or groups who have engaged in violent extremism and/or terrorist activity
  o Family members of radicalised individuals or individuals who have engaged in violent extremism and/or terrorist activity
  o Micro places (e.g., street corners, buildings, police beats, street segments)
  o Macro places (neighbourhoods or larger geographies)

In maps that include systematic reviews, we will visually differentiate between systematic reviews that have included studies and those that do not by the use of colour coding (White et al., 2020).

#### 3.6.3 Dependency

The unit of analysis is the primary study, not the publication or document that reports on the study. We are conscious of the risk of using documents as the unit of analysis, as a visual tool such as an EGM can appear to indicate a preponderance of evidence on an intervention where there are multiple publications drawn from the same research study.

The two most common ways in which dependency can manifest in an EGM are as many: one dependency or as one: many dependency. Firstly, there may be more than one document reporting on the same study (many: one dependency). In this situation, all documents that report on the one study will be linked, and the study is what will appear on the map. Secondly, one document may report on more than one study (one: many dependency). If a document reports on more than one study, we will link that document to both ‘parent’ studies.

Systematic reviews provide a unique challenge in terms of the one: many dependency. A systematic review may contain multiple studies, that may report on multiple interventions and outcome combinations. The challenge lies in deciding how a systematic review should be represented in an EGM. If it were to appear on the same map as its component studies, it would visually suggest more evidence than actually exists. But if it were only to be harvested for its component studies, then the EGM could not show where there has been rigorous synthesis of studies in the area, or attempts at rigorous synthesis, in the case of ‘empty reviews’. We propose a multi‐armed approach to dealing with systematic reviews in the EGM:

1. We will harvest the primary research studies that are included in any systematic review, and link those documents back to the parent study as with any document that has a one:many dependency.
2. We will include systematic reviews in addition to their component studies, *but* systematic reviews will not be visible at the same time as primary studies. We will do this using a strict toggle filter for Study Type, with mutually exclusive options of research synthesis or research studies. This will allow the user to choose between an EGM of primary studies, and an EGM of systematic reviews, to not visually inflate the weight of evidence.

A further form of dependency is where one study compares two eligible treatments head‐to‐head. In these cases, the study itself may be eligible to appear in two different columns of the map. In addition to linking any related documents, we will flag studies of this design so that it is clear where there is the potential for double‐counting. Similarly, if a systematic review is eligible to appear in more than one cell on the map, we will flag the review to ensure that the reader is aware of the potential for double‐counting.

### 3.7 Data collection and analysis

#### 3.7.1 Screening and study selection

Our screening approach will differ slightly for the different sources, but all documents will be assessed on the same final eligibility criteria. Documents from the GPD have already been screened on their full‐text as reporting on a quantitative impact evaluation of a policing intervention. Therefore, we will assess the full‐text of these documents for their eligibility to the review. Documents from other sources have not been verified as quantitative impact evaluations, so we plan to screen these on their title and abstract to remove documents not related to criminal justice responses to terrorism/radicalisation, and then screen eligible full‐text documents on the same criteria as for the GPD data.

For all stages described below, we will develop standardised screening companions and inter‐rater reliability tests to ensure consistency in decision‐making across the research team. For title and abstract screening, each team member will screen the same set of 30 records before commencing screening. Similarly, at the full‐text screening stages, before commencing screening, team members will screen the same set of 30 documents. All team member decisions will be compiled and compared to determine any divergence in decisions. Feedback on screening decisions will be provided with further training when necessary. We will double screen 5% of each screener's excluded documents to identify any potential false‐negative decisions. In instances where a screener's decisions are considered unreliable, their exclusion screenings will be reassigned. Where disagreements about screening decisions occur, these will be mediated by a third team member.

All documents will be screened in DistillerSR—a web based, reference management software that incorporates artificial intelligence (AI) to expedite the review process (Evidence Partners, 2022). DistillerSR has an inbuilt AI function which cross‐checks exclusion decisions. This helps to ensure consistency across reviewers and reduce human error. DistillerSR also draws on machine learning to reorder references based on their likelihood of inclusion, learning from the decisions made by human users (Evidence Partners, 2022). Using this function, we will screen documents up until the point that DistillerSR indicates 100% of all potentially eligible records are included. At this point, we will screen a random sample of 30 additional records. We will repeat this process until no eligible records are identified. We will use the AI function for Title and Abstract screening only.

Following Campbell Collaboration guidance (White et al., 2020), we will screen systematic reviews on their PICOS rather than on their included studies. In this way, we will retain ‘empty reviews', and allow the EGM user to see where synthesis has been attempted but was unsuccessful.

##### GPD sourced documents

First, we will import all documents reporting on a quantitative impact evaluation of a policing intervention captured by the systematic search into DistillerSR. Quantitative impact evaluations indexed in the GPD generally have a pre‐existing full‐text document attached, and document retrieval is therefore not required. We will then screen the full‐text of the records according to the following exclusion criteria:

1. Document is not unique
2. Document is not about a criminal justice intervention for preventing/responding to terrorism/radicalisation.
3. Document does not include an impact evaluation of a criminal justice intervention that aims to prevent or respond to terrorism/radicalisation.

While all efforts are made to remove duplicate records within the GPD, exclusion criterion 1 will be used to remove any that were missed during the initial data cleaning. Exclusion criterion 2 will be used to remove records unrelated to terrorism, radicalisation or extremism. Exclusion criterion 3 will be used to produce a corpus of studies that report on quantitative impact evaluations of an eligible intervention, using an eligible research design.

As part of the GPD protocol, all attempts are made to locate full‐text documents via existing university resources, by ordering documents through the university library, or by directly contacting study authors. Should the search within the GPD return records (i.e., citations) for which we have been unable to locate the full‐text and cannot unequivocally exclude on the title and abstract as not relating to a criminal justice intervention for preventing/responding to terrorism/radicalisation, these documents will be recorded as ‘Studies awaiting classification’.

##### Documents from other sources

Second, we will import all records identified by our electronic searches and other sources (grey literature, hand‐searching, etc.) into DistillerSR. This data will be assessed on title and abstract on the following criteria:

1. Document is not unique
2. Ineligible document type
3. Document is not about a criminal justice intervention for preventing/responding to terrorism/radicalisation.

Before screening, we will make all efforts to remove duplicates and ineligible document types (such as book reviews, blog posts etc). However, criteria 1 and 2 will allow us to remove any that were missed during data cleaning. Exclusion criterion 3 will be used to remove titles and abstracts unrelated to terrorism, radicalisation or extremism.

Records that successfully progress through the title and abstract screening stage will move to a literature retrieval stage. For records without documents, we will endeavour to locate the full‐text document via existing university resources. In instances where we cannot locate the record, we will order the document through the university library or by directly contacting study authors. Documents for which we cannot locate the full‐text and cannot unequivocally exclude on title and abstract will be listed in ‘Studies awaiting classification’.

Once the literature is retrieved, all potentially eligible records will then progress to full‐text eligibility screening. The full‐text of the records according to the following exclusion criteria:

1. Document is not unique
2. Ineligible document type
3. Document does not report on quantitative bivariate or multivariate data which may be indicative of an evaluation
4. Document is not about a criminal justice intervention for preventing/responding to terrorism/radicalisation.
5. Document does not include an impact evaluation of a criminal justice intervention that aims to prevent or respond to terrorism/radicalisation.

The addition of criterion 3 allows us to refine the corpus of studies to those which do not contain bivariate or multivariate data which may be indicative of an impact evaluation. A similar approach is taken for the GPD (Higginson et al., 2015). Criteria 4 and 5 will be used to remove records unrelated to terrorism, radicalisation or extremism and produce a corpus of studies that report on quantitative impact evaluations of an eligible intervention, using an eligible research design.

#### 3.7.2 Data extraction and management

Once deemed eligible for the review, documents will be coded in EPPI‐reviewer. We will use a standardised coding companion (see Supporting Information: Appendix 4) to code documents across the following domains:

1. Participants (sample characteristics, recruitment, etc.)
2. Intervention (setting, components, agencies, etc.)
3. Outcomes (conceptualisation, data source)
4. Research methodology (research design, comparison group)

All coders will code the same four eligible studies to ensure consistency across team members. These will be compiled and compared to determine any divergence in coding, and feedback will be provided before independent coding. To check for inter‐rater reliability, 5% of each person's coding will be double‐coded by a second person. Where disagreements arise, a third review author will be consulted. Study authors will be contacted directly if documents are missing any pertinent information.

#### 3.7.3 Tools for assessing risk of bias/study quality of included studies

Each eligible systematic review in the EGM will be assessed for Risk of Bias using the AMSTAR 2 critical appraisal tool (Shea et al., 2017). Based on ratings across 16 items, the tool generates a rating of overall confidence in the systematic review's results: High (no more than one non‐critical weakness, but no critical flaws); Moderate (more than one non‐critical weakness, but no critical flaws); Low (one critical flaw, with or without non‐critical weaknesses); or Critically Low (more than one critical flaw, with or without non‐critical weaknesses). Two coders will independently code each systematic review using the AMSTAR 2 tool. Any discrepancies between the two coders will be resolved by discussion.

Due to the anticipated size of the EGM, we do not plan to assess confidence in the included primary studies, but note that our methodological thresholds exclude study designs with the highest inherent risk of bias.

#### 3.7.4 Methods for mapping

We will use EPPI‐reviewer to create the EGM (Eppi‐Centre, 2019).

## CONTRIBUTIONS OF AUTHORS

- Content: Michelle Sydes, Lorelei Hine, Laura Dugan, Angela Higginson, Lorraine Mazerolle
- EGM methods: Angela Higginson, Michelle Sydes, Lorelei Hine
- Statistical analysis: Angela Higginson, Michelle Sydes, Lorelei Hine
- Information retrieval: Lorelei Hine, Michelle Sydes, Angela Higginson, Laura Dugan

## DECLARATIONS OF INTEREST

Two of the review authors have internal roles within the Campbell Collaboration Crime and Justice Group. Angela Higginson is an Editor and Co‐chair of the Crime and Justice Coordinating Group (CJCG) and Lorelei Hine is the Managing Editor of the CJCG. Angela Higginson and Lorelei Hine will not be involved in any editorial or internal Campbell Collaboration communications about this review.

## PLANS FOR UPDATING THE EGM

Michelle Sydes will be responsible for updates of this review, which are anticipated to occur every 5 years.

## SOURCES OF SUPPORT

**Internal sources**

• No sources of support provided

**External sources**

• Horizon 2020 (Grant No.: 699824), DHS Science and Technology Directorate, and the Five Research and Development (5RD) Countering Violent Extremism Networks.

## Supporting information

Supporting information.

## References

[cl21273-bib-0002] Bardwell, H. , & Iqbal, M. (2020). The economic impact of terrorism from 2000 to 2018. Peace Economics, Peace Science and Public Policy, 27(2), 227–261. 10.1515/peps-2020-0031

[cl21273-bib-0003] Beutel, A. , & Weinberger, P. (2016). Public‐private partnerships to counter violent extremism: Field principles for action. National Consortium for the Study of Terrorism and Responses to Terrorism.

[cl21273-bib-0004] Borum, R. (2015). Assessing risk for terrorism involvement. Journal of Threat Assessment and Management, 2(2), 63–87. 10.1037/tam0000043

[cl21273-bib-0005] Breen‐Smyth, M. (2014). Theorising the “suspect community”: Counterterrorism, security practices and the public imagination. Critical Studies on Terrorism, 7(2), 223–240. 10.1080/17539153.2013.867714

[cl21273-bib-0006] Carter, J. (2014). Inter‐organisational relationships and law enforcement information sharing post 11 September 2001. Journal of Crime & Justice, 38(4), 522–542. 10.1080/0735648X.2014.927786

[cl21273-bib-0007] Cherney, A. (2021). The release and community supervision of radicalised offenders: Issues and challenges that can influence reintegration. Terrorism and Political Violence, 33(1), 119–137. 10.1080/09546553.2018.1530661

[cl21273-bib-0008] Cherney, A. , & Belton, E. (2021). Evaluating case‐managed approaches to counter radicalization and violent extremism: An example of the proactive integrated support model (PRISM) intervention. Studies in Conflict & Terrorism, 44(8), 625–645. 10.1080/1057610X.2019.1577016

[cl21273-bib-0009] Cherney, A. , & Hartley, J. (2017). Community engagement to tackle terrorism and violent extremism: Challenges, tensions and pitfalls. Policing and Society, 27(7), 750–763. 10.1080/10439463.2015.1089871

[cl21273-bib-0010] Cherney, A. , De Rooy, K. , & Eggins, E. (2021). Mandatory participation in programs to counter violent extremism: A review of evidence for and against. Journal for Deradicalization, 27, 1–33.

[cl21273-bib-0011] Cherney, A. , & Murphy, K. (2016). Being a ‘suspect community' in a post 9/11 world—The impact of the war on terror on Muslim communities in Australia. Australian & New Zealand Journal of Criminology, 49(4), 480–496. 10.1177/0004865815585392

[cl21273-bib-0012] Davis, L. , Helmus, T. , Hunt, P. , Payne, L. , Jahedi, S. , & Tsang, F. (2016). *Assessment of the state and local anti‐terrorism training (SLATT) program*. https://www.rand.org/pubs/research_reports/RR1276.html

[cl21273-bib-0013] Davis, R. , Petersen, K. , Weisburd, D. , & Taylor, B. (2021). Updated protocol: Effects of second responder programs on repeat incidents of family abuse: An updated systematic review and meta‐analysis. Campbell Systematic Reviews, 17(4), e1200. 10.1002/cl2.1200 PMC8988767 36951797

[cl21273-bib-0014] Desmarais, S. , Simons‐Rudolph, J. , Brugh, C. , Schilling, E. , & Hoggan, C. (2017). The state of scientific knowledge regarding factors associated with terrorism. Journal of Threat Assessment and Management, 4(4), 180–209. 10.1037/tam0000090

[cl21273-bib-0015] Dudenhoefer, A. (2018). Resisting radicalisation: A critical analysis of the UK prevent duty. Journal for Deradicalization, 14(1), 154–191.

[cl21273-bib-0016] Eggins, E. , Mazerolle, L. , Higginson, A. , Hine, L. , Walsh, K. , Sydes, M. , McEwan, J. , Hassall, G. , Roetman, S. , Wallis, R. , & Williams, J. (2021). Criminal justice responses to child sexual abuse material offending: A systematic review and evidence and gap map. In Trends & issues in crime and criminal Justice (no. 623). Australian Institute of Criminology. 10.52922/ti78023

[cl21273-bib-0017] Elshimi, M. (2017). De‐radicalisation in the UK prevent strategy: Security, identity and religion. Routledge. 10.4324/9781315271361

[cl21273-bib-0018] Eppi‐Centre . (2019). *EPPI‐Centre Home*. https://eppi.ioe.ac.uk/cms/

[cl21273-bib-0019] Evidence Partners . (2022). *Why organisations choose DistillerSR*. https://www.evidencepartners.com/

[cl21273-bib-0020] Fisher, D. , Dugan, L. , & Chenoweth, E. (2019). Does US presidential rhetoric affect assymmetric political violence? Critical Studies on Terrorism, 12, 132–150. 10.1080/17539153.2018.1494120

[cl21273-bib-0021] Gaibulloev, K. , & Sandler, T. (2019). What we have learned about terrorism since 9/11. Journal of Economic Literature, 57(2), 275–328. 10.1257/jel.20181444

[cl21273-bib-0022] Gielen, A. J. (2019). Countering violent extremism: A realist review for assessing what works, for whom, in what circumstances, and how? Terrorism and Political Violence, 31(6), 1149–1167. 10.1080/09546553.2017.1313736

[cl21273-bib-0023] Glass, G. (1997). Interrupted time series quasi‐experiments. In R. Jaeger (Ed.), Complementary methods for research in education (pp. 589–608). American Educational Research Association.

[cl21273-bib-0024] Haner, M. , Sloan, M. , Cullen, F. , Kulig, T. , & Lero Jonson, C. (2019). Public concern about terrorism: fear, worry, and support for anti‐Muslim policies. Socius, 5, 1–16. 10.1177/2378023119856825

[cl21273-bib-0025] Haner, M. , Sloan, M. M. , Cullen, F. T. , Graham, A. , Lero Jonson, C. , Kulig, T. C. , & Aydın, Ö. (2021). Making America safe again: Public support for policies to reduce terrorism. Deviant Behavior, 42(10), 1209–1227. 10.1080/01639625.2020.1738638

[cl21273-bib-0026] Hardy, K. (2018). Comparing theories of radicalisation with countering violent extremism policy. Journal for Deradicalization, 15, 76–110.

[cl21273-bib-0027] Hardy, K. (2020). A crime prevention framework for CVE. Terrorism and Political Violence, 34(3), 633–659. 10.1080/09546553.2020.1727450

[cl21273-bib-0028] Harris‐Hogan, S. , Barrelle, K. , & Zammit, A. (2015). What is countering violent extremism? Exploring CVE policy and practice in Australia. Behavioral Sciences of Terrorism and Political Aggression, 8(1), 6–24. 10.1080/19434472.2015.1104710

[cl21273-bib-0029] Heath‐Kelly, C. (2013). Counter‐terrorism and the counterfactual: Producing the ‘radicalisation’ discourse and the UK PREVENT strategy. The British Journal of Politics and International Relations, 15(3), 394–415. 10.1111/j.1467-856X.2011.00489.x

[cl21273-bib-0030] Her Majesty's Inspectorate of Probation . (2021). *Extremism and terrorism*. https://www.justiceinspectorates.gov.uk/hmiprobation/research/the-evidence-base-probation/specific-sub-groups/extremism-and-terrorism/

[cl21273-bib-0031] Higginson, A. , Eggins, E. , Mazerolle, L. , & Stanko, E. (2015). *The Global Policing Database* [Database and Protocol. http://www.gpd.uq.edu.au

[cl21273-bib-0032] Horgan, J. (2008). From profiles to pathways and roots to routes: Perspectives from psychology on radicalization into terrorism. The Annals of the American Academy of Political and Social Science, 618(1), 80–94. 10.1177/0002716208317539

[cl21273-bib-0033] Institute for Economics and Peace (IEP) . (2022). *Global terrorism index 2022: Measuring the impact of terrorism*. https://www.visionofhumanity.org/wp-content/uploads/2022/03/GTI-2022-web.pdf

[cl21273-bib-0034] Irwin, N. (2015). The complexity of responding to home‐grown terrorism: Radicalisation, de‐radicalisation and disengagement. Journal of Policing, Intelligence and Counter Terrorism, 10(2), 166–175. 10.1080/18335330.2015.1089639

[cl21273-bib-0035] Jacobs, L. , & van Spanje, J. (2021). Not all terror is alike: How right‐wing extremist and Islamist terror threat affect anti‐immigration party support. International Journal of Public Opinion Research, 33(4), 737–755. 10.1093/ijpor/edaa037

[cl21273-bib-0036] Jensen, M. A. , Atwell Seate, A. , & James, P. A. (2018). Radicalization to violence: A pathway approach to studying extremism. Terrorism and Political Violence, 32(5), 1067–1090. 10.1080/09546553.2018.1442330

[cl21273-bib-0037] Jones, C. , & Narag, R. (2018). Inmate radicalisation and recruitment in prisons. Routledge.

[cl21273-bib-0038] Kerry, A. (2007). *2007‐2008 integrated summative evaluation of the chemical, biological, radiological and nuclear first responder training program*. https://www.publicsafety.gc.ca/cnt/rsrcs/pblctns/archive-vltn-chmcl-blgcl-2007-08/archive-cbrn-pfpi-eng.pdf

[cl21273-bib-0039] Knight, K. (2009). *Exploring the Tampa fusion centre: Interagency collaboration for homeland security*. Retrieved from ProQuest Dissertations and Theses Global database (UMI No.: 3384701).

[cl21273-bib-0040] Kruglanski, A. , Bélanger, J. , & Gunaratna, R. (2019). The three pillars of radicalization: Needs, narratives, and networks. Oxford University Press.

[cl21273-bib-0041] Lamb, J. (2013). Preventing violent extremism; A policing case study of the west midlands. Policing: A Journal of Policy and Practice, 7(1), 88–95. 10.1093/police/pas063

[cl21273-bib-0042] Lewandowski, C. (2012). *Information sharing using a state fusion center: Acase study of the New Jersey Regional Operations Intelligence Centre* (Doctoral dissertation). ProQuest Dissertations and Theses database (UMI No.: 3509012).

[cl21273-bib-0043] Lindekilde, L. , Bertelsen, P. , & Stohl, M. (2016). Who goes, why, and with what effects: The problem of foreign fighters from Europe. Small Wars & Insurgencies, 27(5), 858–877. 10.1080/09592318.2016.1208285

[cl21273-bib-0044] Lum, C. , Kennedy, L. , & Sherley, A. (2006). Are counter‐terrorism strategies effective? The results of the Campbell systematic review on counter‐terrorism evaluation research. Journal of Experimental Criminology, 2(4), 489–516. 10.1007/s11292-006-9020-y

[cl21273-bib-0045] Mazerolle, L. , Cherney, A. , Eggins, E. , Hine, L. , & Higginson, A. (2021). Multiagency programs with police as a partner for reducing radicalisation to violence. Campbell Systematic Reviews, 17(2), e1162. 10.1002/cl2.1162 PMC8356331 37131922

[cl21273-bib-0046] Mazerolle, L. , Eggins, E. , Cherney, A. , Hine, L. , Higginson, A. , & Belton, E. (2020). Police programmes that seek to increase community connectedness for reducing violent extremism behaviour, attitudes and beliefs. Campbell Systematic Reviews, 16(3), e1111. 10.1002/cl2.1111 PMC8356323 37131910

[cl21273-bib-0047] Mazerolle, L. , Eggins, E. , Sydes, M. , Hine, L. , McEwan, J. , Norrie, G. , & Somerville, A. (2018). *Criminal justice responses to domestic and family violence: A rapid review of the literature*. https://www.courts.qld.gov.au/__data/assets/pdf_file/0006/586185/systematic-review-of-criminal-justice-responses-to-domestic-and-family-violence.pdf

[cl21273-bib-0048] Meloy, J. R. (2016). *Terrorist Radicalization Assessment Protocol‐18 (TRAP‐18)*. https://www.rma.scot/wp-content/uploads/2021/04/Terrorist-Radicalization-Assessment-Protocol-18-TRAP-18.pdf 10.1080/00223891.2018.1481077 29927673

[cl21273-bib-0049] Ortiz, C. , Hendricks, N. , & Sugie, N. (2007). Policing terrorism: The response of local police agencies to homeland security concerns. Criminal Justice Studies, 20(2), 91–109. 10.1080/14786010701396830

[cl21273-bib-0050] Pressman, D. , & Flockton, J. (2013). Violent extremist risk assessment: issues and applications of the VERA‐2 in a high‐security correctional setting. In A. Silke (Ed.), Prisons, terrorism and extremism (pp. 122–143). Routledge.

[cl21273-bib-0051] Pundir, P. , Saran, A. , White, H. , Subrahmanian, R. , & Adona, J. (2020). Interventions for reducing violence against children in low‐and middle‐income countries: An evidence and gap map. Campbell Systematic Reviews, 16(4), e1120. 10.1002/cl2.1120 PMC8356324 37016609

[cl21273-bib-0052] Razik, S. , Ehring, T. , & Emmelkamp, P. M. (2013). Psychological consequences of terrorist attacks: Prevalence and predictors of mental health problems in Pakistani emergency responders. Psychiatry Research, 207(1–2), 80–85. 10.1016/j.psychres.2012.09.031 23068079

[cl21273-bib-0053] Ritchie, H. , Hasell, J. , Mathieu, E. , Appel, C. , & Roser, M. (2019). *Terrorism*. Our world in data. https://ourworldindata.org/terrorism

[cl21273-bib-0054] RTI International . (2018). *Countering violent extremism: The application of risk assessment tools in the criminal justice and rehabilitation process*. https://www.dhs.gov/sites/default/files/publications/OPSR_TP_CVE-Application-Risk-Assessment-Tools-Criminal-Rehab-Process_2018Feb-508.pdf

[cl21273-bib-0055] Salguero, J. , Fernández‐Berrocal, P. , Iruarrizaga, I. , Cano‐Vindel, A. , & Galea, S. (2011). Major depressive disorder following terrorist attacks: A systematic review of prevalence, course and correlates. BMC Psychiatry, 11(1), 1–11. 21627850 10.1186/1471-244X-11-96 PMC3120744

[cl21273-bib-0056] Sarma, K. (2017). Risk assessment and the prevention of radicalization from nonviolence into terrorism. American Psychologist, 72(3), 278–288. 10.1037/amp0000121 28383980

[cl21273-bib-0057] Schanzer, D. , Kurzman, C. , Toliver, J. , & Miller, E. (2016). *The challenge and promise of using community policing strategies to prevent violent extremism*. https://nij.ojp.gov/library/publications/challenge-and-promise-using-community-policing-strategies-prevent-violent-0

[cl21273-bib-0058] Shanahan, T. (2016). The definition of terrorism. In P. Jackson (Ed.), Routledge handbook of critical terrorism studies (pp. 103–113). Routledge.

[cl21273-bib-0059] Shea, B. J. , Reeves, B. C. , Wells, G. , Thuku, M. , Hamel, C. , Moran, J. , Moher, D. , Tugwell, P. , Welch, V. , Kristjansson, E. , & Henry, D. A. (2017). AMSTAR 2: A critical appraisal tool for systematic reviews that include randomised or non‐randomised studies of healthcare interventions, or both. BMJ, 21, 358. 10.1136/bmj.j4008 PMC5833365 28935701

[cl21273-bib-0060] Snilstveit, B. , Vojtkova, M. , Bhavsar, A. , Stevenson, J. , & Gaarder, M. (2016). Evidence and gap maps: A tool for promoting evidence informed policy and strategic research agendas. Journal of Clinical Epidemiology, 79, 120–129. 10.1016/j.jclinepi.2016.05.015 27387966

[cl21273-bib-0061] United States Agency for International Development . (2016). *The development response to violent extremism and insurgency*. https://pdf.usaid.gov/pdf_docs/Pdacs400.pdf

[cl21273-bib-0062] van der Heide, L. , & Kearney, O. (2020). *The Dutch approach to extremist offenders*. https://icct.nl/app/uploads/2020/02/vanderheide-kearney-thedutchapproach.pdf?msclkid=e9c39505aa8d11eca4825dec4a43bd8f

[cl21273-bib-0063] Vejvodová, P. , & Kolář, O. (2020). Training prison staff to recognize inmate radicalisation. Security Journal, 33(4), 552–564. 10.1057/s41284-019-00192-8

[cl21273-bib-0064] White, H. , Albers, B. , Gaarder, M. , Kornør, H. , Littell, J. , Marshall, Z. , Mathew, C. , Pigott, T. , Snilstveit, B. , Waddington, H. , & Welch, V. (2020). Guidance for producing a Campbell evidence and gap map. Campbell Systematic Reviews, 16(4), e1125:1–27. 10.1002/cI2.1125 PMC8356343 37016607

[cl21273-bib-0065] Williams, M. J. , Horgan, J. G. , & Evans, W. P. (2016). Evaluation of a multi‐faceted, U.S. community‐based, Muslim‐led CVE program. US Department of Justice.

[cl21273-bib-0066] Williamson, H. (2019). Pride and prejudice: Exploring how identity processes shape public attitudes towards Australian counter‐terrorism measures. Australian & New Zealand Journal of Criminology, 52(4), 558–577. 10.1177/0004865819846944

[cl21273-bib-0067] Williamson, H. , & Murphy, K. (2020). Animus toward Muslims and its association with public support for punitive counter‐terrorism policies: Did the Christchurch terrorist attack mitigate this association? Journal of Experimental Criminology, 18, 343–363. 10.1007/s11292-020-09450-x

[cl21273-bib-0068] Wilner, A. , & Dubouloz, C. (2010). Homegrown terrorism and transformative learning: An interdisciplinary approach to understanding radicalization. Global Change, Peace & Security, 22(1), 33–51. 10.1080/14781150903487956

[cl21273-bib-0069] Windisch, S. , Simi, P. , Ligon, G. , & McNeel, H. (2016). Disengagement from ideologically‐based and violent organizations: A systematic review of the literature. Journal for Deradicalization, 9, 1–38.

[cl21273-bib-0070] Wolfowicz, M. , Litmanovitz, Y. , Weisburd, D. , & Hasisi, B. (2019). A field‐wise systematic review and meta‐analysis of putative risk and protective factors for radicalization outcomes. Journal of Quantitative Criminology, 36, 407–447. 10.1007/s10940-019-09439-4

