# Supplementary material for: PROTOCOL: Criminal justice interventions for preventing terrorism and radicalisation: An evidence and gap map
Source: Campbell Syst Rev. 2022 Sep 1;18(3):e1273. doi: 10.1002/cl2.1273 (PMC9436177; doi:10.1002/cl2.1273)
Supplement: Supplementary file 1 — Supporting information. [file CL2-18-e1273-s001.docx]

# Appendices

## 1 Appendix A: GPD Systematic search strategy

### Search Terms

To ensure optimum sensitivity and specificity, the GPD search strategy utilises a combination of free-text and controlled vocabulary search terms. Because controlled vocabularies and search capabilities vary across databases, the exact combination of search terms and field codes are adapted to each database. Final search syntax for each location will be reported in the final review.

The free-text search terms for the GPD are provided in Table 1 and are grouped by substantive (i.e., some form of policing) and evaluation terminology. Although the search strategy may vary slightly across search locations, it follows a number of general rules:

- Search terms are combined into search strings using Boolean operators “AND” and “OR”. Specifically, terms within each category are combined with “OR”, and categories will be combined with “AND”. For example: (police OR policing OR “law#enforcement”) AND (analy* OR ANCOVA OR ANOVA OR …).
- Compound terms (e.g., law enforcement) are considered single terms in search strings by using quotation marks (i.e., “law*enforcement”) to ensure that the database searches for the entire term rather than separate words.
- Wild cards and truncation codes are used for search terms with multiple iterations from a stem word (e.g., evaluation, evaluate) or spelling variations (e.g., evaluat* or randomi#e).
- If a database has a controlled vocabulary term that is equivalent to “POLICE”, the term is combined in a search string that includes both the policing and evaluation free-text search terms. This approach ensures that the search retrieves documents that do not use policing terms in the title/abstract but have been indexed as being related to policing in the database.  An example of this approach is the following search string:  (((SU: “POLICE”) OR (TI,AB,KW: police OR policing OR “law*enforcement”)) AND (TI,AB,KW: intervention* OR evaluat* OR compar* OR …)).
- For search locations with limited search functionality, a broad search that uses only the policing free-text terms is implemented.
- Multidisciplinary database searches are limited to relevant disciplines (e.g., include social sciences but exclude physical sciences).
- Search results are refined to exclude specific types of documents that are not suitable for systematic reviews (e.g., newspapers, front/back matter, book reviews).

####

#### Table 1. Free-text search terms for the GPD systematic search

| **Policing Search Terms** | **Evaluation Search Terms** | | | |
| --- | --- | --- | --- | --- |
| police  policing  “law*enforcement”  constab*  detective*  sheriff* | analy*  ANCOVA  ANOVA  “ABAB design”  “AB design”  baseline  causa*  “chi#square”  coefficient*  “comparison condition*”  “comparison group*”  “control condition*”  “control group*”  correlat*  covariat*  “cross#section*” | data  effect*  efficacy  eval*  experiment*  hypothes*  impact*  intervent*  interview*  longitudinal  MANCOVA  MANOVA  “matched group”  measure*  “meta-analy*”  “odds#ratio* | outcome*  paramet*  “post-test”  posttest  “post test”  predict*  “pre-test”  pretest  program*  “propensity score*”  quantitative  “quasi#experiment*”  questionnaire*  random*  RCT  regress* | result*  “risk#ratio*”  sampl*  “standard deviation*”  statistic*  studies  study  survey*  “systematic review*”  “t#test*”  “time#series”  treatment*  variable*  variance |

### Search Locations

To reduce publication and discipline bias, the GPD search strategy adopts an international scope and involves searching for literature across a number of disciplines (e.g., criminology, law, political science, public health, sociology, social science and social work). The search captures a comprehensive range of published (i.e., journal articles, book chapters, books) and unpublished literature (e.g., working papers, governmental reports, technical reports, conference proceedings, dissertations) by implementing a search strategy across bibliographic/academic, grey literature, and dissertation databases or repositories.

It is noted that there is substantial overlap of the content coverage between many of the databases. Therefore, the *Optimal Searching of Indexing Databases* (OSID) computer program (Neville & Higginson, 2014) has been used to analyse the content crossover for all databases that have accessible content coverage lists. OSID analyses the content coverage and creates a search location solution that provides the most comprehensive coverage via the least number of databases. Another advantage of using OSID when designing a search strategy is the reduction in the number of duplicates that would need to be removed prior to the screening phase. Databases with >10 unique titles are searched in full, whereas databases with ≤10 unique titles were searched only the unique titles and any non-serial content (e.g., reports, conference proceedings). Where a modified search of a database would be more labour-intensive than a full search and export results, a full search of the database is conducted. The final search locations and solutions are reported in Table 2.

#### Table 2. GPD search locations and protocol (January 1^st^ 1950 – December 2019)

| **INDEXED & ACADEMIC DATABASES** |  | **CONTENT COVERAGE FED INTO OSID?** | **FULL OR MODIFIED SEARCH?** | **SEARCH MODIFICATIONS** |
| --- | --- | --- | --- | --- |
| **ProQuest** | Criminal Justice | Yes | Full | None. |
|  | Dissertation and Theses Database Global | Not Available | Modified | Social Sciences subset. |
|  | Political Science | Yes | Full | None. |
|  | Periodical Archive Online | Yes | Full | None. |
|  | Research Library | Yes | Modified | Social Sciences subset. |
|  | Social Science Journals | Yes | Full | None. |
|  | Sociology | Yes | Modified | Search 2 unique journal titles and non-serial content only. |
|  | Applied Social Sciences Index and Abstracts | Yes | Full | None. |
|  | International Bibliography of the Social Sciences | Yes | Full | None. |
|  | Public Affairs Information Service | Yes | Full | None. |
|  | Social Services Abstracts | Yes | Modified | Search 5 unique journal titles and non-serial content only. |
|  | Sociological Abstracts | Yes | Full | None. |
|  | Worldwide Political Sciences Abstracts | Yes | Modified | Search 9 unique journal titles and non-serial content only. |
| **EBSCO** | Academic Search Premier | Yes | Full | None. |
|  | Criminal Justice Abstracts | Yes | Full | None. |
|  | EconLit | Yes | Full | None. |
|  | MEDLINE with Full-Text (for initial search 1950-2014) | Yes | Full | None. |
|  | Social Sciences Full-Text | Yes | Full | None. |
| **OVID** | International Political Science Abstracts | Not Available | Full | None. |
|  | PsycARTICLES | Yes | Modified | Search 4 unique journal titles only. |
|  | PsycEXTRA | Not Available | Full | None. |
|  | PsycINFO | Yes | Full | None. |
|  | Social Work Abstracts | Not Available | Full | None. |
| **Web of Science** | Current Contents Connect – Social and Behavioural Sciences Edition | Yes | Modified | Search 1 unique journal title and non-serial content only. |
|  | Book Citation Index (Social Sciences and Humanities) | Not Available | Full | None. |
|  | Conference Proceedings Citation Index (Social Sciences and Humanities) | Not Available | Full | None. |
|  | MEDLINE with Full-Text (for searches 2015 onwards) | Yes | Full | None. |
|  | Social Science Citation Index | Yes | Full | None. |
| **Informit** | Australian Attorney General Information Service | Yes | Full | None. |
|  | Australian Criminology Database (CINCH) | Yes | Full | None. |
|  | Australian Federal Police Database | Yes | Full | None. |
|  | Australian Public Affairs Full-Text | Yes | Full | None. |
|  | DRUG | Yes | Full | None. |
|  | Health & Society Database | Yes | Modified | Search unique journal titles and non-serial content only. |
|  | Humanities and Social Sciences Collection | Yes | Full | None. |
| **Gale-Cengage** | Expanded Academic ASAP | Yes | Full | None. |
| **STANDALONE & OPEN ACCESS DATABASES** | Cambridge Journals Online | Yes | Modified | Search 4 unique journal titles in Law and Political Science collections and full search of Social Studies collection. |
|  | Directory of Open Access Journals | Yes | Full | None. |
|  | HeinOnline | Yes | Modified | Law Journals Online collection only. |
|  | JSTOR | Yes | Modified | Search unique titles across the Law, Political Science, Public Health, Public Policy, Social Work and Sociology collections only. The Criminal Justice collection had no unique content and so will be excluded from the search. Only 10% of content in this database have abstracts and a full-text search returns >250,000 results because of inability to construct complex search strings. Therefore, a modified search of the unique titles across these collections will be more pragmatic than a full search of the database. |
|  | Oxford Scholarship Online | Yes | Full | None. |
|  | Sage Journals Online and Archive (Sage Premier) | Yes | Modified | Search 5 unique journal titles and non-serial content only. |
|  | ScienceDirect | Yes | Full | None. |
|  | SCOPUS | Yes | Full | None. |
|  | SpringerLink | Yes | Full | Although this database has low uniqueness when combined with the full set of databases, a full search using only the policing search terms will be more pragmatic than a modified search on unique titles because of the restricted search functionality of this database. |
|  | Taylor & Francis Online | Yes | Modified | Although this database has low uniqueness when combined with the full set of databases, a full search using only the policing search terms will be more pragmatic than a modified search on unique titles because of the restricted search functionality of this database. |
|  | Wiley Online Library | Yes | Full | None. |
|  | California Commission on Peace Officer Standards & Training Library | No | Full | None. |
|  | Cochrane Library | No | Full | None. |
|  | CrimeSolutions.gov | No | Full | None. |
|  | Database of Abstracts of Reviews of Effectiveness (DARE) | No | Full | None. |
|  | FBI – The Fault (Reports and Publications) | No | Full | None. |
|  | Evidence-Based Policing Matrix | No | Full | None. |
|  | International Initiative for Impact Evaluation Database (3ie) | No | Full | None. |
|  | National Criminal Justice Reference Service | No | Full | None. |
|  | Safety Lit Database | No | Full | None. |
|  | Australian Institute of Criminology | No | Full | None. |
|  | Bureau of Police Research and Development (India) | No | Full | None. |
|  | Canadian Police Research Catalogue | No | Full | None. |
|  | Centre for Problem-Oriented Policing | No | Full | None. |
|  | College of Policing (including POLKA and Crime Reduction Toolkit) | No | Full | None. |
|  | European Police College (CEPOL) | No | Full | None. |
|  | Evidence for Policy and Practice Information and Coordinating Centre | No | Full | None. |
|  | National Research Institute of Police Science (Japanese) | No | Full | None. |
|  | Office of Community Oriented Policing Services | No | Full | None. |
|  | Police Executive Research Forum (US) | No | Full | None. |
|  | Police Foundation (US) | No | Full | None. |
|  | Tasmania Institute of Law Enforcement Studies (Australia) | No | Full | None. |
|  | Policing Online Information System (POLIS, Europe) | No | Full | None. |
|  | Scottish Institute for Policing Research | No | Full | None. |
|  | Centre of Excellence in Policing and Security (Australian, now archived) | No | Full | None. |
|  | Alcohol and Alcohol Problems Science Database (ETOH, now archived) | No | Full | None. |
|  | African Journals Online | No | Full | None. |
|  | Campbell Collaboration Library of Systematic Reviews | No | Full | None. |
|  | Criminal Justice Press (Crime Prevention Studies, volumes 1-27) | No | Full | None. |
|  | Danish National Police (Politi) | No | Full | None. |
|  | Drug Policy Alliance – Lindesmith Library (Online Resource Library) | No | Full | None. |
|  | DrugScope | No | Full | None. |
|  | Finnish Police (Poliisi) | No | Full | None. |
|  | GeoRef | No | Full | None. |
|  | German Federal Criminal Police Office (Bundeskriminalamt) | No | Full | None. |
|  | Home Office (United Kingdom) | No | Full | None. |
|  | Indian Citation Index (Social Science and Humanities Subset) | No | Full | None. |
|  | Institute for Law and Justice | No | Full | None. |
|  | Jill Dando Institute of Crime Science (JDI) | No | Full | None. |
|  | Justice Research and Statistics Association- State Statistical Analysis Centers | No | Full | None. |
|  | Ministry of Justice (United Kingdom) | No | Full | None. |
|  | Netherlands Institute for the Study of Crime and Law Enforcement (NSCR) | No | Full | None. |
|  | Netherlands Police (Politie) | No | Full | None. |
|  | New Zealand Ministry of Justice | No | Full | None. |
|  | New Zealand Police | No | Full | None. |
|  | Norwegian Ministry of Justice and the Police | No | Full | None. |
|  | Royal Canadian Mounted Police | No | Full | None. |
|  | SAGE Knowledge | No | Full | None. |
|  | Swedish National Council on Crime Prevention (Brå) | No | Full | None. |
|  | Swedish Police Service | No | Full | None. |
|  | Urban Institute | No | Full | None. |
|  | YU-DSpace Repository | No | Full | None. |

## 2 Appendix B: GPD Systematic Compilation Strategy

### Inclusion Criteria

Each record captured by the GPD systematic search must satisfy all inclusion criteria to be included in the GPD: timeframe, intervention and research design. There are no restrictions applied to the types of outcomes, participants, settings or languages considered eligible for inclusion in the GPD.

#### Types of interventions

Each document must contain an impact evaluation of a policing intervention. Policing interventions are defined as some kind of a strategy, program, technique, approach, activity, campaign, training, directive, or funding/organisational change that involves police in some way (other agencies or organisations can be involved). Police involvement is broadly defined as:

- Police initiation, development or leadership
- Police are recipients of the intervention or the intervention is related, focused or targeted to police practices
- Delivery or implementation of the intervention by police

#### Types of study designs

The GPD includes quantitative impact evaluations of policing interventions that utilise randomised experimental (e.g., RCTs) or quasi-experimental evaluation designs with a valid comparison group that does not receive the intervention. The GPD includes designs where the comparison group receives ‘business-as-usual’ policing, no intervention or an alternative intervention (treatment-treatment designs).

The specific list of research designs included in the GPD are as follows:

- Systematic reviews with or without meta-analyses
- Cross-over designs
- Cost-benefit analyses
- Regression discontinuity designs
- Designs using multivariate controls (e.g., multiple regression)
- Matched control group designs with or without pre-intervention baseline measures (propensity or statistically matched)
- Unmatched control group designs with pre-post intervention measures which allow for difference-in-difference analysis
- Unmatched control group designs without pre-intervention measures where the control group has face validity
- Short interrupted time-series designs with control group (less than 25 pre- and 25 post-intervention observations)
- Long interrupted time-series designs with or without a control group (≥25 pre- and post-intervention observations)
- Raw unadjusted correlational designs where the variation in the level of the intervention is compared to the variation in the level of the outcome

The GPD excludes single group designs with pre- and post-intervention measures as these designs are highly subject to bias and threats to internal validity.

### Systematic Screening

To establish eligibility, records captured by the GPD search are progress through a series of systematic stages which are summarised in Table 1, with additional detail provided in the following subsections.

All research staff working on the GPD undergo standardised training before beginning work within any of the stages detailed below. Staff then complete short training simulations to enable an assessment of their understanding of the GPD protocols and highlight any areas for additional training. In addition, random samples of each staff’s work are regularly cross-checked to ensure adherence to protocols. Disagreements about screening decisions between staff are mediated by either the project manager or GPD chief investigators.

#### Title and abstract screening

After removing duplicates, the title and abstract of records captured by the GPD systematic search is screened by trained research staff to identify potentially eligible research that satisfies the following criteria:

- Document is dated between 1950 – present
- Document is unique (i.e., not a duplicate)
- Document is about police or policing
- Document is an eligible document type (e.g., not a book review)

Records are excluded if the answer to any one of the criteria is unambiguously ‘No’, and will be classified as potentially eligible otherwise. Records classified as eligible at the title and abstract screening stage progress to full-text document retrieval and screening stages.

#### Full-text eligibility screening

Wherever possible, a full-text electronic version of an eligible record is imported into *SysReview*(review management software; Higginson & Neville, 2015). For records without an electronic version, a hardcopy of the record is located to enable full-text eligibility screening. The full-text of each document is screened to identify studies that satisfy the following criteria:

- Document is dated between 1950 – present
- Document is unique
- Document reports a quantitative statistical comparison
- Document reports on policing evaluation
- Document reports in a quantitative impact evaluation of a policing intervention
- Evaluation uses an eligible research design

| **SYSTEMATIC SEARCH OF PUBLISHED & UNPUBLISHED LITERATURE** |
| --- |
| ⇓ |
| **EXPORT SEARCH RESULTS**   - Bibliographic data and abstracts exported into EndNote - Data cleaned and duplicate records removed |
| ⇓ |
| **IMPORT SEARCH RESULTS INTO *SYSREVIEW*** |
| ⇓ |
| **SCREEN TITLES AND ABSTRACTS FOR ELIGIBILITY**   1. Not a duplicate document? 2. Between 1950 – present? 3. About police or policing? 4. Eligible document type?   ***If not clearly excluded on any criteria…*** |
| ⇓ |
| **DOCUMENT RETRIEVAL**   - Retrieve electronic and hard copies of all eligible documents - Attach electronic versions to records in *SysReview* |
| ⇓ |
| **SCREEN FULL-TEXT OF DOCUMENTS**  **FOR FINAL ELIGIBILITY**   1. Not a duplicate document? 2. Between 1950 – present? 3. Quantitative statistical comparison? 4. Policing intervention? 5. Quantitative impact evaluation? 6. Eligible research design?   ***If ‘Yes’ to all…*** |
| ⇓ |
| **CATEGORISE ELIGIBLE DOCUMENTS**   1. Research design 2. Intervention location 3. Publication date 4. Problem targeted 5. Evaluation outcome measure(s) 6. Type of policing intervention |
| ⇓ |
| **GLOBAL POLICING DATABASE (GPD)**  Web-based  Searchable  Updated biennially |

*Table 1.* GPD systematic compilation process

## 3 Appendix C: Example search syntax

Search syntax for Criminal Justice Abstracts (via EBSCO)

| **#** | **Query** | **Limiters/Expanders** | **Last Run Via** | **Results** |
| --- | --- | --- | --- | --- |
| S5 | S1 AND S2 AND S3 | Limiters - Publication Date: 20020101-20211231; Document Type: Abstract, Article, Book, Book Chapter, Case Study, Essay, Excerpt, Opinion, Other, Proceeding, Report Search modes - Boolean/Phrase | Interface - EBSCOhost Research Databases Search Screen - Advanced Search Database - Criminal Justice Abstracts | 2,761 |
| S4 | S1 AND S2 AND S3 | Search modes - Boolean/Phrase | Interface - EBSCOhost Research Databases Search Screen - Advanced Search Database - Criminal Justice Abstracts | 3,183 |
| S3 | TI ( “comparison condition*” OR “comparison-condition*” OR “comparison group*” OR “comparison-group*” OR “control condition*” OR “control-condition*” OR “control group*” OR “control-group*” OR effective OR efficac* OR evaluat* OR experiment* OR intervent* OR “matched group*” OR “matched-group*” OR program* OR “quasi-experiment*” OR “quasiexperiment*” OR “quasi experiment*” OR random* OR RCT OR treatment* OR trial* ) OR AB ( “comparison condition*” OR “comparison-condition*” OR “comparison group*” OR “comparison-group*” OR “control condition*” OR “control-condition*” OR “control group*” OR “control-group*” OR effective OR efficac* OR evaluat* OR experiment* OR intervent* OR “matched group*” OR “matched-group*” OR program* OR “quasi-experiment*” OR “quasiexperiment*” OR “quasi experiment*” OR random* OR RCT OR treatment* OR trial* ) OR KW ( “comparison condition*” OR “comparison-condition*” OR “comparison group*” OR “comparison-group*” OR “control condition*” OR “control-condition*” OR “control group*” OR “control-group*” OR effective OR efficac* OR evaluat* OR experiment* OR intervent* OR “matched group*” OR “matched-group*” OR program* OR “quasi-experiment*” OR “quasiexperiment*” OR “quasi experiment*” OR random* OR RCT OR treatment* OR trial* ) | Search modes - Boolean/Phrase | Interface - EBSCOhost Research Databases Search Screen - Advanced Search Database - Criminal Justice Abstracts | 193,333 |
| S2 | ( accused OR acquit* OR adjourn* OR adjudicat* OR *admiss* OR affida* OR appeal* OR appellate OR apprehend* OR arbitrat* OR arraign* OR *arrest* OR attorney* OR authorit* OR bail* OR barrister* OR breach* OR “case manage*” OR “case-manage*” OR caution* OR charge* OR clerk* OR confinement* OR convict* OR coroner* OR correction* OR court* OR crime* OR criminal* OR “cross examin*” OR “cross-examin*” OR custod* OR defendant* OR defense OR defence OR detain* OR detention* OR deter* OR divert* OR diversion* OR enforc* OR execut* OR felon* OR forensic* OR gaol* OR guilt* OR “high security” OR “high-security” OR “halfway house” OR “halfway-house” OR *imprison* OR incarcerat* OR indict* OR infract* OR infring* OR injunct* OR inquest* OR innocen* OR inmate* OR juris* OR jail* OR judge* OR judic* OR juror* OR juries OR jury OR justice OR law* OR legal* OR legislat* OR litigat* OR “low security” OR “low-security” OR magistrate* OR mandat* OR mitigat* OR marshal* OR misdem* OR “medium security*” OR “medium-security*” OR offend* OR offence* OR officer* OR official* OR ordinance OR parole* OR pardon* OR penal* OR plea* OR precedent* OR prevent* OR prison* OR probat* OR prohibit* OR prosecut* OR punish*OR recividis* OR rehab* OR reintegrat* OR remand* OR reoffend* OR “re-offend*” OR ruling* OR sanction* OR sentenc* OR solicitor* OR statut* OR subpoena* OR supervis* OR surveil* OR suspect* OR testif* OR testimon* OR *trial* OR tribunal* OR verdict* OR victim*OR witness* ) OR ( accused OR acquit* OR adjourn* OR adjudicat* OR *admiss* OR affida* OR appeal* OR appellate OR apprehend* OR arbitrat* OR arraign* OR *arrest* OR attorney* OR authorit* OR bail* OR barrister* OR breach* OR “case manage*” OR “case-manage*” OR caution* OR charge* OR clerk* OR confinement* OR convict* OR coroner* OR correction* OR court* OR crime* OR criminal* OR “cross examin*” OR “cross-examin*” OR custod* OR defendant* OR defense OR defence OR detain* OR detention* OR deter* OR divert* OR diversion* OR enforc* OR execut* OR felon* OR forensic* OR gaol* OR guilt* OR “high security” OR “high-security” OR “halfway house” OR “halfway-house” OR *imprison* OR incarcerat* OR indict* OR infract* OR infring* OR injunct* OR inquest* OR innocen* OR inmate* OR juris* OR jail* OR judge* OR judic* OR juror* OR juries OR jury OR justice OR law* OR legal* OR legislat* OR litigat* OR “low security” OR “low-security” OR magistrate* OR mandat* OR mitigat* OR marshal* OR misdem* OR “medium security*” OR “medium-security*” OR offend* OR offence* OR officer* OR official* OR ordinance OR parole* OR pardon* OR penal* OR plea* OR precedent* OR prevent* OR prison* OR probat* OR prohibit* OR prosecut* OR punish*OR recividis* OR rehab* OR reintegrat* OR remand* OR reoffend* OR “re-offend*” OR ruling* OR sanction* OR sentenc* OR solicitor* OR statut* OR subpoena* OR supervis* OR surveil* OR suspect* OR testif* OR testimon* OR *trial* OR tribunal* OR verdict* OR victim*OR witness* ) OR ( accused OR acquit* OR adjourn* OR adjudicat* OR *admiss* OR affida* OR appeal* OR appellate OR apprehend* OR arbitrat* OR arraign* OR *arrest* OR attorney* OR authorit* OR bail* OR barrister* OR breach* OR “case manage*” OR “case-manage*” OR caution* OR charge* OR clerk* OR confinement* OR convict* OR coroner* OR correction* OR court* OR crime* OR criminal* OR “cross examin*” OR “cross-examin*” OR custod* OR defendant* OR defense OR defence OR detain* OR detention* OR deter* OR divert* OR diversion* OR enforc* OR execut* OR felon* OR forensic* OR gaol* OR guilt* OR “high security” OR “high-security” OR “halfway house” OR “halfway-house” OR *imprison* OR incarcerat* OR indict* OR infract* OR infring* OR injunct* OR inquest* OR innocen* OR inmate* OR juris* OR jail* OR judge* OR judic* OR juror* OR juries OR jury OR justice OR law* OR legal* OR legislat* OR litigat* OR “low security” OR “low-security” OR magistrate* OR mandat* OR mitigat* OR marshal* OR misdem* OR “medium security*” OR “medium-security*” OR offend* OR offence* OR officer* OR official* OR ordinance OR parole* OR pardon* OR penal* OR plea* OR precedent* OR prevent* OR prison* OR probat* OR prohibit* OR prosecut* OR punish*OR recividis* OR rehab* OR reintegrat* OR remand* OR reoffend* OR “re-offend*” OR ruling* OR sanction* OR sentenc* OR solicitor* OR statut* OR subpoena* OR supervis* OR surveil* OR suspect* OR testif* OR testimon* OR *trial* OR tribunal* OR verdict* OR victim*OR witness* ) | Search modes - Boolean/Phrase | Interface - EBSCOhost Research Databases Search Screen - Advanced Search Database - Criminal Justice Abstracts | 502,919 |
| S1 | TI ( extremis* OR "far left*" OR "far-left*" OR "far right*" OR “far-right*” OR "foreign fight*" OR "foreign-fight*" OR "freedom fight*" OR "freedom-fight*" OR guerrilla OR "homeland security" OR "ideological violence*" OR "ideologically motivat*" OR "ideologically-motivat*" OR indoctrinat* OR "left wing*" "left-wing*" OR "lone wol*" OR "lone-wol*"OR militant* OR "national security" OR "political violence*" OR "politically motivat*" OR "politically-motivat*" OR radicali* OR rebel* OR "religious violence*" OR "religiously motivat*" OR "religiously-motivat*" OR "right wing*" OR "right-wing*" OR "single issue*" OR "single-issue*" OR supremacis* OR terror* OR vigilante* OR vigilantism OR deradicali* OR “de-radicali*" OR "counter-terror*" OR counterterror* OR "counter-extremis*" OR counterextremis* OR separatis* OR militia* OR jihad* ) OR AB ( extremis* OR "far left*" OR "far-left*" OR "far right*" OR “far-right*” OR "foreign fight*" OR "foreign-fight*" OR "freedom fight*" OR "freedom-fight*" OR guerrilla OR "homeland security" OR "ideological violence*" OR "ideologically motivat*" OR "ideologically-motivat*" OR indoctrinat* OR "left wing*" "left-wing*" OR "lone wol*" OR "lone-wol*"OR militant* OR "national security" OR "political violence*" OR "politically motivat*" OR "politically-motivat*" OR radicali* OR rebel* OR "religious violence*" OR "religiously motivat*" OR "religiously-motivat*" OR "right wing*" OR "right-wing*" OR "single issue*" OR "single-issue*" OR supremacis* OR terror* OR vigilante* OR vigilantism OR deradicali* OR “de-radicali*" OR "counter-terror*" OR counterterror* OR "counter-extremis*" OR counterextremis* OR separatis* OR militia* OR jihad* ) OR KW ( extremis* OR "far left*" OR "far-left*" OR "far right*" OR “far-right*” OR "foreign fight*" OR "foreign-fight*" OR "freedom fight*" OR "freedom-fight*" OR guerrilla OR "homeland security" OR "ideological violence*" OR "ideologically motivat*" OR "ideologically-motivat*" OR indoctrinat* OR "left wing*" "left-wing*" OR "lone wol*" OR "lone-wol*"OR militant* OR "national security" OR "political violence*" OR "politically motivat*" OR "politically-motivat*" OR radicali* OR rebel* OR "religious violence*" OR "religiously motivat*" OR "religiously-motivat*" OR "right wing*" OR "right-wing*" OR "single issue*" OR "single-issue*" OR supremacis* OR terror* OR vigilante* OR vigilantism OR deradicali* OR “de-radicali*" OR "counter-terror*" OR counterterror* OR "counter-extremis*" OR counterextremis* OR separatis* OR militia* OR jihad* ) | Search modes - Boolean/Phrase | Interface - EBSCOhost Research Databases Search Screen - Advanced Search Database - Criminal Justice Abstracts | 19,240 |

## 4 Appendix C: Coding Form

Coding form[1]

### General Study Details

1. Study ID [textbox] (this will refer to the document/s using a unique identifier)
2. Report ID [textbox] (this will be used to denote whether multiple studies are nested within the document, e.g., Smith 2020a, 2020b, etc.)
3. What type of document is this study? [dropdown menu]
   1. Peer-reviewed journal article
   2. Book chapter
   3. Dissertation
   4. Conference presentation
   5. Government report, technical report, or working paper
   6. Other (specify in textbox)
4. Where was the intervention implemented (please list city, county, state/province and country as applicable)? [textbox]
5. In what year was the intervention implemented? [textbox]
6. If the evaluation and/or intervention was funded, record the funding source. [textbox]
7. Publication status
8. Ongoing
9. Completed

### Participants

1. Who are the participants? [*checkboxes*]
   1. Criminal justice practitioners
   2. Victims
   3. Radicalised individuals or groups (including pre-criminal justice involved radicalised individuals)
   4. Individuals or groups who have engaged in violent extremism and/or terrorist activity
   5. Individuals or groups who have been identified as at-risk of becoming radicalised or engaging in violent extremism and/or terrorist activity
   6. Family members of radicalised individuals or individuals who have engaged in violent extremism and/or terrorist activity
   7. Communities
   8. Micro places (e.g., street corners, buildings, police beats, street segments)
   9. Macro places (neighbourhoods or larger geographies)
   10. Other
2. What were the eligibility criteria for inclusion in the study? [textbox]

| **Number of Participants** | **Treatment** | **Comparison** | **Total** |
| --- | --- | --- | --- |
| Referred to study |  |  |  |
| Consented |  |  |  |
| Assigned |  |  |  |
| Began intervention |  |  |  |
| Completed intervention |  |  |  |
| Completed follow-up 1 |  |  |  |
| Completed follow-up 2 (if applicable) |  |  |  |

1. Describe the characteristics of the sample. [textboxes]

|  | **Treatment** | **Comparison** | **Total** |
| --- | --- | --- | --- |
| Age (M, SD, range) |  |  |  |
| Gender (% female) |  |  |  |
| Ethnicity (proportions) |  |  |  |
| Socioeconomic status (proportions) |  |  |  |

1. Record any other pertinent sample information for both the treatment and comparison groups. [textbox].

### Methodological Details and Nature of Comparisons

1. General research design classification [dropdown menu]
   1. Systematic review
   2. Randomised controlled trial
   3. Quasi-experiment
   4. Other (specify in textbox)
2. What type of comparison condition was used? [dropdown menu]
   1. No treatment
   2. Treatment-as-usual (specify in textbox)
   3. Alternative treatment (specify in textbox)
   4. Waitlist control
   5. Other (specify in textbox)

### Intervention Details

1. What is the name of the intervention(s), as reported by study authors? [textbox]
2. What settings were used during the intervention(s)? [select all that apply]
   1. Prisons/correctional facilities
   2. Courts
   3. Community
   4. School
   5. Workplace
   6. Places of worship (e.g., church)
   7. Home
   8. Other (specify)
3. Which agencies were involved in implementing the intervention? [select all that apply]
   1. Police
   2. Prison/correctional facilities
   3. Courts
   4. Probation/parole
   5. Other criminal justice agency not listed here [specify]
   6. Non-criminal justice partner* must be selected in combination with a-d options
4. If multiagency, how many agencies were involved? [textbox]
5. If the intervention involved non-criminal justice partner/s, please specify who these were [textbox]
6. Does the intervention target terrorism, radicalisation, or both? [textbox]
7. What level of prevention does the intervention involve (e.g., primary, secondary, tertiary)? [textbox]
8. Describe the intervention provided to participants, ensuring you record the specific components or materials implemented and the mode of implementation. [textbox]

### Outcome(s) Measurement*

*To be completed for each eligible outcome within a study (or group of reports for a study).

1. Briefly describe the outcome being measured (e.g., its name, how it is conceptualised by study authors, what higher or lower value mean). [textbox]
2. What category does the outcome fall within? [dropdown menu]
   1. Extremism or radicalisation
   2. Crime or offending
   3. Psychosocial
   4. Other  (specify)
3. How was the outcome data gathered? [dropdown menu]
   1. Self-report
   2. Observation
   3. Official source
   4. Interview
   5. Other (specify in textbox)

[1] This form has been informed by published coding forms (e.g., Littel et al., 2008; Mazerolle, Higginson, & Eggins, in press; Mitchell, Wilson, Eggers, & MacKenzie, 2012; Mazerolle et al., 2021).
